# Marrying Blood Relations

**Published:** 1869-05

**Authors:** 


### MARRYING BLOOD RELATIONS.

A few years ago Count Trepeni married his sister's daughter. On any day at this present writing, these grandchildren of the Duchess of Tuscany, and scions of the House of Bourbon, may be seen playing in the garden, pale, ugly and ghastly, with their ankles supported by iron splints and bands. A few years ago, in our own street, one of the loveliest of women, in person, character and domestic accomplishments, married her cousin; they have but one child, now four years old; large scabs break out on its body every season; it has never spoken a word, and has never been well for an hour since its birth. These things have happened where there has been no relationship between the married, but they so much oftener occur as a result of blood marriages that it has become a commonly recognised fact, and those who are wise will act in accordance with it.

It is very true that in the first ages of the world, near blood relations were necessarily married; but that necessity ceased after a few generations. Then, an entire out door life, plainness of living, and other circumstances, were antagonizers of harmful results. We are not living under the circumstances of patriarchal and shepherd life; we have to do with the present, and must deal with facts as they present themselves to us. No man ought to be willing to run even a remote risk of transmitting a life-long blemish of bodily depravity, of constitution or mental infirmity to the child of his bosom—he has no right to do so, for it is an irreparable wrong to those who may be born to him.

